# Pan-Asian subgroup analysis of EV-302/KEYNOTE-A39: a phase 3 study to evaluate enfortumab vedotin and pembrolizumab in patients with untreated advanced urothelial carcinoma

**DOI:** 10.1007/s10147-025-02950-8

**Published:** 2026-01-21

**Authors:** Eiji Kikuchi, Michiel S. Van der Heijden, Begoña P. Valderrama, Shilpa Gupta, Jens Bedke, Sang Joon Shin, Jian-Ri Li, Jun Guo, Pongwut Danchaivijitr, Ravindran Kanesvaran, Se Hoon Park, Wen-Pin Su, Shuya Kandori, Woo Kyun Bae, Alvin Wong, Seema Gorla, Abhishek Bavle, Xuesong Yu, Yi-Tsung Lu, Thomas Powles

**Affiliations:** 1 St. Marianna University School of Medicine, Kanagawa, Japan; 2 The Netherlands Cancer Institute, Amsterdam, The Netherlands; 3 Hospital Universitario Virgen del Rocío, Seville, Spain; 4 Taussig Cancer Institute, Cleveland Clinic Foundation, Cleveland, OH USA; 5 Department of Urology & Transplantation Surgery, Eva Mayr-Stihl Cancer Center, Klinikum Stuttgart, Germany; 6 Severance Hospital, Yonsei University Health System, Seoul, South Korea; 7 Taichung Veterans General Hospital, Taichung, Taiwan; 8 Beijing Cancer Hospital, Beijing, China; 9 Siriraj Hospital, Mahidol University, Bangkok, Thailand; 10 National Cancer Centre Singapore, Singapore, Singapore; 11 Samsung Medical Center, Sungkyunkwan University School of Medicine, Seoul, Korea; 12 Department of Oncology, College of Medicine, National Cheng Kung University Hospital, National Cheng Kung University, Tainan, Taiwan; 13 Institute of Clinical Medicine, College of Medicine, National Cheng Kung University, Tainan, Taiwan; 14 University of Tsukuba, Ibaraki, Japan; 15 Chonnam National University Medical School & Hwasun Hospital, Hwasun, South Korea; 16 National University Cancer Institute, Singapore, Singapore; 17 Astellas Pharma Inc, Northbrook, IL USA; 18 Merck & Co., Inc, Rahway, NJ USA; 19 Pfizer Inc, New York, NY USA; 20 Barts Cancer Institute, Queen Mary University of London, London, UK

**Keywords:** Enfortumab vedotin, Pembrolizumab, Urothelial cancer, Asia, Phase III

## Abstract

**Background:**

In the phase 3 EV-302 study, enfortumab vedotin–pembrolizumab (EV + P) significantly prolonged overall survival (OS) and progression-free survival (PFS) versus chemotherapy in patients with untreated locally advanced/metastatic urothelial carcinoma (la/mUC). We present a post hoc analysis in a pan-Asian population.

**Methods:**

Patients from China, Japan, Singapore, South Korea, Taiwan, and Thailand received 3-week cycles of EV (1.25 mg/kg; intravenously; Days 1 and 8) plus P (200 mg; intravenously; Day 1) or chemotherapy (gemcitabine [Days 1 and 8] plus cisplatin/carboplatin [Day 1]). Primary endpoints were PFS and OS. Secondary endpoints included objective response rate (ORR) and safety.

**Results:**

Overall, 176 patients were included (EV + P, *n* = 94; chemotherapy, *n* = 82). Median follow-up was 28.9 months for EV + P recipients and 26.6 months for chemotherapy recipients. EV + P prolonged PFS and OS versus chemotherapy, reducing the risk of disease progression or death by 63% (hazard ratio [HR], 0.37; 95% confidence interval [CI], 0.24–0.57) and death by 67% (HR, 0.33; [95% CI, 0.20–0.54]), respectively. Confirmed ORR was 72.2% versus 35.0%. Grade ≥ 3 treatment-related adverse events occurred in 66.0% of EV + P recipients and 68.4% of chemotherapy recipients. Most commonly maculopapular rash (11.7%) and hyperglycemia (10.6%) for EV + P and neutropenia (25.0%), anemia (19.7%), and neutrophil count decreased (18.4%) for chemotherapy.

**Conclusion:**

EV + P demonstrated a clinically meaningful survival benefit in Asian patients with untreated la/mUC, with no new safety signals observed, consistent with the global EV-302 study. Results support guideline recommendations for EV + P as preferred first-line therapy in la/mUC.

**Clinical trial registration:**

NCT04223856 (registered January 8, 2020).

**Graphical abstract:**

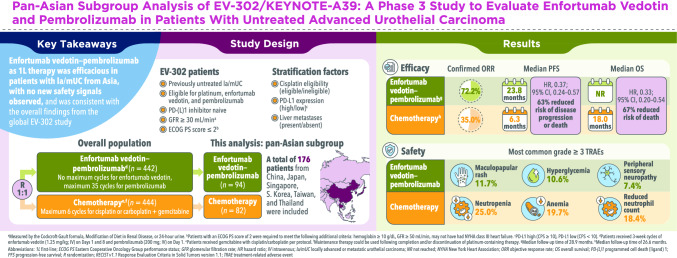

**Supplementary Information:**

The online version contains supplementary material available at 10.1007/s10147-025-02950-8.

## Introduction

Bladder cancer poses a major public health challenge in East Asia and carries a high economic and societal burden due to increased age-standardized incidence and disability-adjusted life years [1]. In 2020, the number of new cases and deaths from bladder cancer was highest in East Asia (132,316 new cases; 54,206 deaths) [2]. Upper tract urothelial carcinoma is also prevalent in this region [3].

Locally advanced or metastatic urothelial carcinoma (la/mUC) has a poor outcome, with a 5-year overall survival (OS) rate of ~ 5%; for comparison, OS is approximately 9 to 15 months following treatment with standard first-line (1L) platinum-based chemotherapy [4, 5]. Pembrolizumab, a programmed cell death protein-1 (PD-1) inhibitor, is approved in the United States and other regions as 1L therapy for platinum-ineligible patients with la/mUC and in Europe for cisplatin-ineligible patients whose tumors have a combined positive score (CPS) of programmed death ligand-1 (PD-L1) ≥ 10 [6, 7]. Pembrolizumab has demonstrated a clinically meaningful survival benefit and durable response in platinum-ineligible patients with la/mUC [6, 8, 9]. However, the combination of chemotherapy and immune checkpoint inhibitors (ICI) other than nivolumab has not demonstrated a consistent OS benefit for patients with la/mUC [10–12].

Enfortumab vedotin is a first-in-class antibody–drug conjugate (ADC) composed of a fully human monoclonal antibody targeting Nectin-4 conjugated via a cleavable linker to monomethyl auristatin E (MMAE), a microtubule-disrupting agent that induces cell death [13, 14]. Enfortumab vedotin monotherapy was first approved in the US for the treatment of cisplatin-ineligible patients with la/mUC who have previously received ≥ 1 prior lines of therapy or a PD-1/PD-L1 inhibitor and platinum-containing chemotherapy. Approval was based on the phase 3 EV-301 study (NCT03474107), reporting an objective response rate (ORR) of 40.6% for enfortumab vedotin monotherapy versus 17.9% for chemotherapy, with a manageable safety profile [15]. Enfortumab vedotin in combination with pembrolizumab is considered standard of care 1L treatment for la/mUC per treatment guidelines and recommendations [13, 16, 17]. Approval was based on results from phase 3 EV-302/KEYNOTE-A39, in which enfortumab vedotin–pembrolizumab nearly doubled OS (median, 31.5 months versus 16.1 months, respectively; *P* < 0.001) and progression-free survival (PFS; median, 12.5 months versus 6.3 months, respectively; *P* < 0.001) versus platinum-based chemotherapy in patients with la/mUC, irrespective of cisplatin eligibility and PD-L1 expression status [18]. Updated 1-year data from the EV-302 study demonstrated continued superior efficacy and durability of response versus chemotherapy [19].

Data from the post hoc subgroup analysis of a pan-Asian population (China, Japan, Singapore, South Korea, Taiwan, and Thailand) from the global EV-302 study are presented.

## Materials and methods

### Study design and patients

This exploratory analysis evaluates patients from China, Japan, Singapore, South Korea, Taiwan, and Thailand enrolled in the global EV-302 study, the design and primary analysis of which have been previously described [18]. Briefly, EV-302 was a phase 3, global, open-label randomized trial evaluating enfortumab vedotin–pembrolizumab versus platinum-based chemotherapy in patients with previously untreated la/mUC. Patients were randomly assigned 1:1 to receive 3-week cycles of enfortumab vedotin (1.25 mg/kg, intravenously [IV]) on Days 1 and 8 plus pembrolizumab (200 mg, IV) on Day 1, or gemcitabine (1000 mg/m^2^, IV) on Days 1 and 8 and either cisplatin (70 mg/m^2^, IV) or carboplatin (area under the curve 4.5 or 5 mg/mL/min according to local guidelines, IV) on Day 1 of each 3-week cycle. Patients were stratified according to cisplatin eligibility (eligible or ineligible), PD-L1 expression status (high [CPS ≥ 10] or low [CPS < 10]), and liver metastases (present or absent). Eligible adults had histologically confirmed, unresectable, previously untreated la/mUC with measurable disease per Response Evaluation Criteria in Solid Tumors (RECIST) v1.1; an Eastern Cooperative Oncology Group (ECOG) performance status score of 0 to 2; and adequate baseline hematologic, hepatic, and renal function.

### Endpoints

Dual primary endpoints were PFS by blinded independent central review (BICR) according to RECIST v1.1 and OS. Select secondary endpoints included ORR, duration of response (DOR), disease control rate (DCR) assessed by BICR and investigators, PFS by investigator assessment per RECIST v1.1, and safety.

### Statistical analysis

Hazard ratio (HR) and corresponding 95% confidence interval (CI) from the stratified Cox proportional hazards regression model are presented. Median survival was estimated using the Kaplan–Meier (KM) method and reported with estimated KM curves and corresponding 95% CIs by treatment group. DOR was summarized descriptively by KM methods for patients with a confirmed response (complete response [CR] or partial response [PR] per RECIST v1.1). Patients who received maintenance therapy as the first subsequent therapy in the chemotherapy group were not censored for PFS and DOR analyses. ORR and DCR were estimated for each treatment group. Safety was analyzed in patients who had received ≥ 1 dose of any study treatment.

## Results

### Patients

**Fig. 1:**
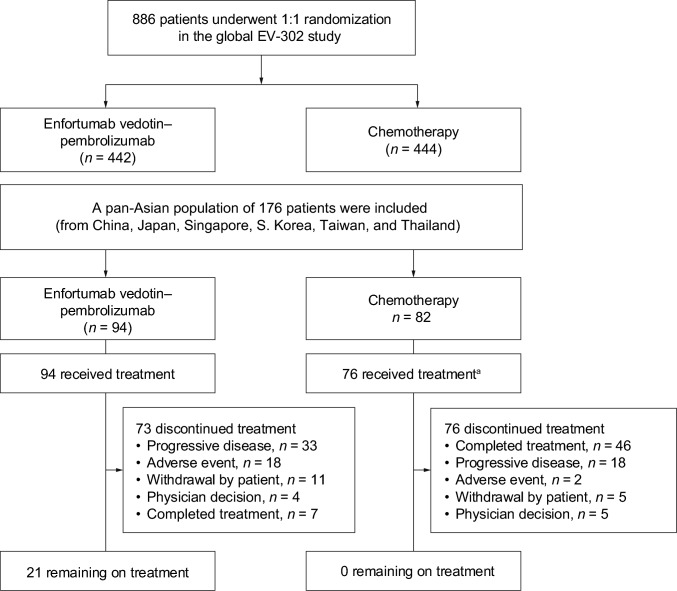
Patient disposition: CONSORT. Data cutoff date was August 8, 2024. ^a^Six patients in the chemotherapy group did not receive treatment due to withdrawal of consent. *CONSORT* Consolidated Standards of Reporting Trials

The data cutoff date was August 8, 2024. The pan-Asian subgroup included 176 patients of Asian ethnicity, with 2 patients in China, 40 in Japan, 5 in Singapore, 58 in South Korea, 51 in Taiwan, and 20 in Thailand. Patients were assigned to receive either enfortumab vedotin–pembrolizumab (*n* = 94) or chemotherapy (*n* = 82). In the enfortumab vedotin–pembrolizumab group, all patients received treatment, and 21 (22.3%) patients remained on treatment at data cutoff (Fig. 1). In the chemotherapy group, 76 patients received treatment, with a maximum of 6 cycles, and none remained on treatment at data cutoff. In the enfortumab vedotin–pembrolizumab group, the primary reasons for treatment discontinuation were progressive disease (33 [35.1%]) and adverse events (AEs; 18 [19.1%]). In the chemotherapy group, the primary reasons for treatment discontinuation were treatment completion (46 [56.1%]) and progressive disease (18 [22.0%]).

Baseline patient demographic and disease characteristics were generally balanced between the two groups. In the enfortumab vedotin–pembrolizumab group, the median (range) age was 69.5 (37–86) years, and 66.0% of the patients were men. In the chemotherapy group, the median (range) age was 68.0 (48–91) years, and 72.0% of the patients were men. In the enfortumab vedotin–pembrolizumab group, median (range) H-score for Nectin-4 was 285.0 (2–300); PD-L1 expression data were available for 93 patients, of which 52 (55.9%) showed PD-L1 high expression (CPS ≥ 10). In the chemotherapy group, median (range) H-score for Nectin-4 was 281.5 (0–300); 45 (54.9%) patients showed PD-L1 high expression (CPS ≥ 10) (Table 1). Approximately half of the patients in both groups were eligible to receive cisplatin-based therapy. In the enfortumab vedotin–pembrolizumab group, 21 (22.3%) patients received subsequent systemic therapy; of these patients, 18 (19.1%) received platinum-based therapy and 2 (2.1%) received other therapies. In the chemotherapy group, 54 (65.9%) patients received subsequent systemic therapy, 8 (9.8%) patients received platinum-based therapy, and 42 (51.2%) patients received ICIs either as maintenance or second-line therapy (**Online Resource 1**).

**Table 1:** Patient demographic and baseline clinical characteristics: pan-Asian subgroup

Characteristic	Enfortumab vedotin–pembrolizumab (*n* = 94)	Chemotherapy (*n* = 82)
Male, *n* (%)	62 (66.0)	59 (72.0)
Female, *n* (%)	32 (34.0)	23 (28.0)
Age, median (range), years	69.5 (37–86)	68.0 (48–91)
ECOG PS score,* n* (%)		
0	57 (60.6)	40 (48.8)
1	36 (38.3)	40 (48.8)
2	1 (1.1)	2 (2.4)
Smoking status, *n* (%)		
Former or current smoker	49 (52.1)	39 (47.6)
Nonsmoker	45 (47.9)	42 (51.2)
Unknown	0	1 (1.2)
Primary tumor location, *n* (%)		
Upper tract	49 (52.1)	43 (52.4)
Lower tract	45 (47.9)	39 (47.6)
Cisplatin eligibility^a^, *n* (%)		
Eligible	46 (48.9)	46 (56.1)
Ineligible	48 (51.1)	36 (43.9)
Disease status, *n* (%)		
Metastatic	89 (94.7)	76 (92.7)
Locally advanced	5 (5.3)	6 (7.3)
Metastatic category^b^, *n* (%)		
Visceral metastases	70 (74.5)	55 (67.1)
Bone	14 (14.9)	17 (20.7)
Liver	10 (10.6)	14 (17.1)
Lung	41 (43.6)	32 (39.0)
Lymph node–only disease	19 (20.2)	22 (26.8)
Nectin-4 H-score		
Patients with evaluable tumor tissue, *n*	85	78
Median (range)	285.0 (2–300)	281.5 (0–300)
PD-L1 expression^c^, *n/n* (%)		
PD-L1 high (CPS ≥ 10)	52/93 (55.9)	45/82 (54.9)
PD-L1 low (CPS < 10)	41/93 (44.1)	37/82 (45.1)

^a^Cisplatin eligibility was based on post-randomization corrections of CRF

^b^A patient may have had metastatic disease in more than one location

^c^CPS status was determined using the validated PD-L1 IHC 22C3 PharmDx assay at Neogenomics and Labcorp. One patient in the enfortumab vedotin–pembrolizumab group had a sample of inadequate tissue quality for analysis

*CPS* combined positive score; *CRF* case report form; *ECOG* *PS* Eastern Cooperative Oncology Group performance status; *IHC* immunohistochemistry; *PD-L1* programmed death ligand-1

### Efficacy

The median (range) duration of treatment with enfortumab vedotin was 9.4 (0.6–38.0) months, with a median (range) of 12 (1–49) cycles received. For pembrolizumab, median (range) duration of treatment was 12.6 (0.6–30.1) months, with a median (range) of 17.5 (1–35) cycles received. In the chemotherapy group, the median (range) duration of treatment was 4.1 (0.7–5.3) months, with a median (range) of 6 (1–6) cycles received.

**Fig. 2:**
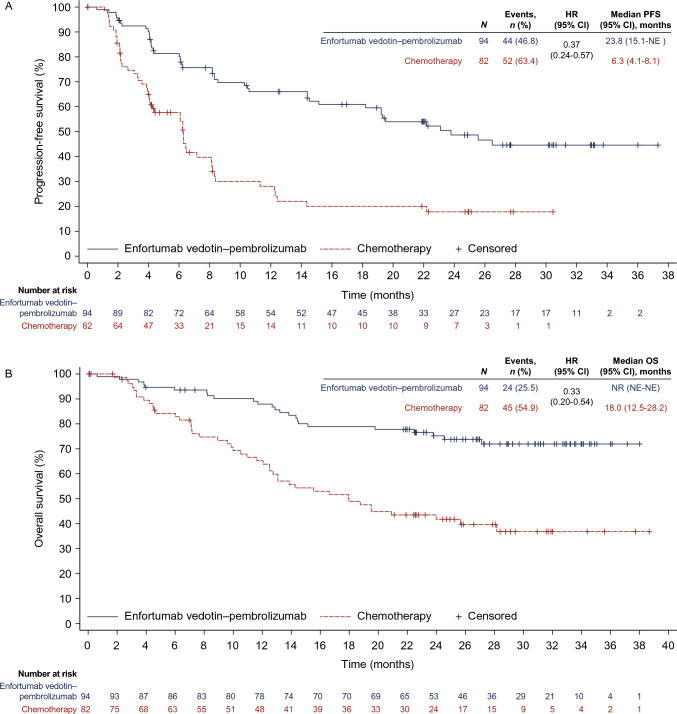
Kaplan–Meier plot for the pan-Asian population for progression-free survival by BICR **A** and overall survival **B**. *BICR* blinded independent central review; *CI* confidence interval; *HR* hazard ratio; *NE* not estimable; *NR* not reached; *OS* overall survival; *PFS* progression-free survival

The risk of disease progression or death was 63% lower in the enfortumab vedotin–pembrolizumab group versus the chemotherapy group (HR, 0.37; 95% CI, 0.24–0.57). Treatment with enfortumab vedotin–pembrolizumab resulted in longer PFS than treatment with chemotherapy. The median duration of PFS in the enfortumab vedotin–pembrolizumab group was 23.8 months (95% CI, 15.1–not estimable) and in the chemotherapy group was 6.3 months (95% CI, 4.1–8.1). The proportion of patients with disease progression or death was 46.8% (44/94) in the enfortumab vedotin–pembrolizumab group and 63.4% (52/82) in the chemotherapy group (Fig. 2A).

At data cutoff, median follow-up was 28.9 months in the enfortumab vedotin–pembrolizumab group and 26.6 months in the chemotherapy group. Overall, 69 deaths occurred, including 24 of 94 (25.5%) patients in the enfortumab vedotin–pembrolizumab group and 45 of 82 (54.9%) patients in the chemotherapy group. The risk of death was 67% lower in the enfortumab vedotin–pembrolizumab group versus the chemotherapy group (HR, 0.33; 95% CI, 0.20–0.54). Median OS in the enfortumab vedotin–pembrolizumab group was not estimable and was 18.0 months (95% CI, 12.5–28.2) in the chemotherapy group (Fig. 2B).

The confirmed ORR was higher in the enfortumab vedotin–pembrolizumab group than in the chemotherapy group (72.2% [95% CI, 61.8–81.1] versus 35.0% [95% CI, 24.7–46.5]; Table 2). CR was observed in 37 of 90 (41.1%) patients in the enfortumab vedotin–pembrolizumab group and in 14 of 80 (17.5%) patients in the chemotherapy group. The median (95% CI) DOR in the enfortumab vedotin–pembrolizumab group was not reached and was 13.9 (9.3–not estimable) months in the chemotherapy group. (Table 2).

**Table 2:** Summary of response; response-evaluable set^a^

Response	Enfortumab vedotin–pembrolizumab (*n* = 90)	Chemotherapy (*n* = 80)
Confirmed ORR, *n* (%) (95% CI)^b^	65 (72.2) (61.8–81.1)	28 (35.0) (24.7–46.5)
Best overall response^c^, *n* (%)		
Complete response	37 (41.1)	14 (17.5)
Partial response	28 (31.1)	14 (17.5)
Stable disease	18 (20.0)	27 (33.8)
Progressive disease	5 (5.6)	18 (22.5)
Not evaluable^d^	0	2 (2.5)
No assessment^e^	2 (2.2)	5 (6.3)
Median DOR (95% Cl), months	NR (21.2–NE)	13.9 (9.3–NE)

^a^ORR was analyzed in the response-evaluable set, which included all patients randomly assigned with measurable disease per RECIST v1.1 at baseline

^b^Computed using the Clopper-Pearson method (Clopper 1934)

^c^Best overall response according to RECIST v1.1 by BICR. CR or PR was confirmed with repeat scans ≥ 28 days after initial response

^d^Patients had post-baseline assessment, and the best overall response was determined not evaluable per RECIST v1.1

^e^Patients had no post-baseline response assessment

*BICR* blinded independent central review; *CI* confidence interval; *CR* complete response; *DOR* duration of response; *NE* not estimable; *NR* not reached; *ORR* objective response rate; *PR* partial response; *RECIST v1.1* Response Evaluation Criteria in Solid Tumors version 1.1

### Safety

Treatment-related AEs (TRAEs) of any grade occurred in 93 of 94 patients (98.9%) in the enfortumab vedotin–pembrolizumab group and in 70 of 76 patients (92.1%) in the chemotherapy group (Table 3). The most common TRAEs of any grade (≥ 25% of patients in either arm) in the enfortumab vedotin–pembrolizumab group were peripheral sensory neuropathy (54.3%), pruritus (53.2%), alopecia (44.7%), rash maculopapular (27.7%), and decreased appetite (25.5%). The most common TRAEs in the chemotherapy group were anemia (48.7%), nausea (34.2%), neutropenia (30.3%), thrombocytopenia, decreased appetite, neutrophil count decreased (each 26.3%), and fatigue (25.0%) (Table 3). Grade ≥ 3 TRAEs occurred in 62 of 94 (66.0%) patients in the enfortumab vedotin–pembrolizumab group and in 52 of 76 (68.4%) of patients in the chemotherapy group. The most common grade ≥ 3 TRAEs were rash maculopapular (11.7%), hyperglycemia (10.6%), and peripheral sensory neuropathy (7.4%) in the enfortumab vedotin–pembrolizumab group and neutropenia (25.0%), anemia (19.7%), and neutrophil count decreased (18.4%) in the chemotherapy group (Table 3).

**Table 3:** Treatment-related adverse events; safety analysis set^a^

Adverse event, *n* (%)	Enfortumab vedotin–pembrolizumab (*n* = 94)		Chemotherapy (*n* = 76)	
Any-grade TRAEs	93 (98.9)		70 (92.1)	
Grade ≥ 3 TRAEs	62 (66.0)		52 (68.4)	
TRAEs leading to death^b^	2 (2.1)		0	
TRAEs leading to withdrawal of any treatment	33 (35.1)		5 (6.6)	
TRAEs leading to withdrawal of enfortumab vedotin	27 (28.7)		–	
TRAEs leading to withdrawal of pembrolizumab	18 (19.1)		–	
TRAEs in ≥ 15% of patients in either arm	Any grade	Grade ≥ 3	Any grade	Grade ≥ 3
Peripheral sensory neuropathy	51 (54.3)	7 (7.4)	10 (13.2)	0
Pruritus	50 (53.2)	3 (3.2)	7 (9.2)	0
Alopecia	42 (44.7)	0	10 (13.2)	0
Rash maculopapular	26 (27.7)	11 (11.7)	4 (5.3)	0
Decreased appetite	24 (25.5)	3 (3.2)	20 (26.3)	2 (2.6)
Fatigue	20 (21.3)	4 (4.3)	19 (25.0)	5 (6.6)
Alanine aminotransferase increased	19 (20.2)	2 (2.1)	2 (2.6)	0
Diarrhea	19 (20.2)	2 (2.1)	4 (5.3)	0
Nausea	19 (20.2)	1 (1.1)	26 (34.2)	4 (5.3)
Neutropenia	9 (9.6)	6 (6.4)	23 (30.3)	19 (25.0)
Anemia	8 (8.5)	2 (2.1)	37 (48.7)	15 (19.7)
Thrombocytopenia	7 (7.4)	0	20 (26.3)	8 (10.5)
Neutrophil count decreased	5 (5.3)	3 (3.2)	20 (26.3)	14 (18.4)
Weight decreased	18 (19.1)	1 (1.1)	4 (5.3)	0
Aspartate aminotransferase increased	16 (17.0)	3 (3.2)	1 (1.3)	1 (1.3)
Hyperglycemia	16 (17.0)	10 (10.6)	0	0
Dysgeusia	15 (16.0)	0	2 (2.6)	0
Rash papular	15 (16.0)	2 (2.1)	2 (2.6)	0
Hypothyroidism	15 (16.0)	2 (2.1)	1 (1.3)	0
Eczema	15 (16.0)	3 (3.2)	0	0
Platelet count decreased	0	0	13 (17.1)	9 (11.8)

^a^The safety analysis set included all patients randomly assigned who received at least 1 dose of study drug (or any component of combination therapy)

^b^The investigator assessed treatment relatedness, including causality to any study drug. One patient died due to asthenia and 1 patient due to pneumonitis

*TRAE* treatment-related adverse event

In the enfortumab vedotin–pembrolizumab group, the most common grade ≥ 3 TRAEs of special interest associated with enfortumab vedotin were skin reactions (28.7%), hyperglycemia (10.6%), and peripheral neuropathy (8.5%) (**Online Resource 2**). In the enfortumab vedotin–pembrolizumab group, the most common grade ≥ 3 treatment-emergent AEs of special interest for pembrolizumab were severe skin reactions (18.1%), pneumonitis (7.4%), and hypothyroidism (2.1%) (**Online Resource 3**). TRAEs resulting in discontinuation of any treatment occurred in 33 (35.1%) and 5 (6.6%) patients in the enfortumab vedotin–pembrolizumab and chemotherapy groups, respectively. In the enfortumab vedotin–pembrolizumab group, TRAEs led to the discontinuation of enfortumab vedotin in 27 (28.7%) patients and of pembrolizumab in 18 (19.1%) patients; 2 patients died due to a TRAE in the enfortumab vedotin–pembrolizumab group (1 each for asthenia and pneumonitis) (Table 3).

## Discussion

In the EV-302 study, enfortumab vedotin–pembrolizumab showed superior efficacy versus chemotherapy in a broad population and is now considered standard of care for patients with la/mUC [18]. In the pan-Asian subgroup of the global EV-302 study, patients with previously untreated la/mUC demonstrated a clinically meaningful survival benefit when treated with enfortumab vedotin−pembrolizumab versus chemotherapy.

In the pan-Asian subgroup, baseline demographic and disease characteristics were generally balanced between groups and consistent with the overall EV-302 population, although a few differences were noted. Compared with the overall EV-302 study population, the pan-Asian subgroup had a numerically higher proportion of patients who were non-smokers and with upper tract tumors and a lower proportion of patients with liver metastases; these differences were not tested for statistical significance versus the previously published global study population [18]. The higher proportion of upper tract tumors in the pan-Asian subgroup is consistent with previous findings in patients with la/mUC in Asia [20, 21].

The median duration of treatment with enfortumab vedotin–pembrolizumab was numerically longer in the pan-Asian subgroup than in the global EV-302 study (14.8 versus 9.4 months, respectively), which may have impacted the efficacy of enfortumab vedotin–pembrolizumab in this subgroup population compared with the global EV-302 population [18]. Despite a higher proportion of patients with upper tract la/mUC (a poor prognostic factor) in the pan-Asian subgroup, PFS and OS improvements were consistent with the global EV-302 population. In the pan-Asian subgroup, enfortumab vedotin–pembrolizumab reduced the risk of disease progression by 63% versus chemotherapy; in the global EV-302 population, the risk of disease progression or death was 55% lower (HR, 0.45; 95% Cl, 0.38–0.54) with enfortumab vedotin–pembrolizumab versus chemotherapy. Similarly, in the pan-Asian subgroup, the risk of death was 67% lower with enfortumab vedotin−pembrolizumab versus chemotherapy; in the global EV-302 population, the risk of death was 53% lower (HR, 0.47; 95% Cl, 0.38–0.58) with enfortumab vedotin–pembrolizumab versus chemotherapy [18]. The proportion of patients with confirmed ORR in this subgroup analysis was twice as high with enfortumab vedotin−pembrolizumab than with chemotherapy (72.2% versus 35.0%, respectively), which is consistent with the ORR observed in the global EV-302 population (67.7% versus 44.4%) [18]. Notably, the CR rate was 41.1% in the pan-Asian subgroup compared with 17.5% with chemotherapy.

In the global phase 3 CheckMate 901 study, compared with gemcitabine–cisplatin alone, nivolumab plus gemcitabine–cisplatin improved outcomes in patients with previously untreated unresectable or metastatic urothelial carcinoma. The risk of disease progression or death was 28% lower (HR, 0.72; 95% CI, 0.59–0.88) and the risk of death was 22% lower (HR, 0.78; 95% CI, 0.63–0.96) with nivolumab plus gemcitabine–cisplatin [10]. In the Asian subgroup analysis of the global CheckMate 901 population, the risk of disease progression or death was 47% lower (HR, 0.53; 95% CI, 0.32–0.88) and the risk of death was 31% lower (HR, 0.69; 95% CI, 0.42–1.15) with nivolumab plus gemcitabine–cisplatin [22]. The ORR with nivolumab plus gemcitabine–cisplatin versus gemcitabine–cisplatin was 58.3% versus 39.3% in the Asian subgroup, which is consistent with the global CheckMate 901 population (57.6% versus 43.1%, respectively) [10, 22].

The recently published results of the phase 3 RC48-C016 trial demonstrated the improved efficacy of disitamab vedotin, a human epidermal growth factor receptor 2 (HER2)-ADC, in combination with toripalimab, a PD-1 monoclonal antibody, compared with chemotherapy in patients with HER2-expressing la/mUC [23]. The interpretation of these data in the context of the current European Society for Medical Oncology clinical practice guidelines and NCCN Clinical Practice Guidelines in Oncology (NCCN Guidelines^®^) for bladder cancer, which recommend enfortumab vedotin–pembrolizumab as the preferred 1L therapy for la/mUC [17, 24], is limited due to several differences in the RC48-C016 and EV-302 study designs. The RC48-C016 trial enrolled patients with HER2-expressing la/mUC only and was exclusively conducted in China, where avelumab maintenance therapy was not available. In contrast, EV-302 was a global, international study that did not require any biomarker selection and included several Asian regions where avelumab maintenance therapy was available [18].

During enrollment for EV-302, the JAVELIN Bladder 100 study was published, reporting improved OS with avelumab maintenance in patients with la/mUC whose disease had not progressed with platinum-based chemotherapy [25]. Thus, in the EV-302 study, patients could receive avelumab maintenance therapy if deemed eligible by the investigator. In the pan-Asian population of the EV-302 study, 20.7% of patients in the chemotherapy group received avelumab therapy and 30.4% of patients in the global EV-302 chemotherapy group received avelumab therapy as their first subsequent systemic therapy [18]. This was likely because avelumab was not reimbursed in a significant portion of Asia, including Korea and Tawain, during the study [26, 27] and the real-world data that showed that < 1/3 of patients who received platinum-based chemotherapy subsequently received avelumab [28–30]. Nevertheless, the possibility that subsequent avelumab therapy may have confounded OS comparisons should be considered.

The safety profile of enfortumab vedotin–pembrolizumab was consistent with previous findings [31] and aligned with the EV-302 safety results [18]. Enfortumab vedotin–related AEs of special interest were generally consistent with those in the overall population except for grade ≥ 3 skin reactions and hyperglycemia, which were numerically higher in the pan-Asian subgroup [18]. However, AEs were generally manageable with dose modifications and the incidence of certain AEs did not affect the treatment discontinuation rate. AEs of special interest for pembrolizumab were also consistent with those in the overall population except for pneumonitis, which was numerically higher in the pan-Asian subgroup [18]. Notably, the higher incidence of AEs of special interest observed in the pan-Asian subgroup may be related to the longer median duration of treatment with enfortumab vedotin–pembrolizumab compared with the global EV-302 study.

Results from this subgroup analysis should be interpreted cautiously as this analysis was exploratory and not adequately powered to make firm conclusions. In addition, patients from the different pan-Asian countries in this analysis were unevenly distributed, with some countries, such as China and Singapore, contributing a small number of patients. This may limit the robustness and generalizability of these results in these countries.

This subgroup analysis of the global EV-302/KEYNOTE-A39 study showed that enfortumab vedotin in combination with pembrolizumab prolonged PFS and OS versus chemotherapy in Asian patients with previously untreated la/mUC. The safety profile was generally manageable, with no new safety signals identified for either enfortumab vedotin or pembrolizumab. These findings align with those from the broader patient population of EV-302, demonstrating consistent benefit of enfortumab vedotin–pembrolizumab across multiple geographic regions and subgroups. Together, the results support the enfortumab vedotin and pembrolizumab combination as the preferred 1L treatment for patients with la/mUC.

## Ethics approval

The study protocol and all amendments were approved by the institutional review board or ethics committee at each study site, and the study was conducted in accordance with the principles of the Declaration of Helsinki, Good Clinical Practice guidelines as defined by the International Council for Harmonisation, and all applicable regulatory requirements.

## Informed consent

All patients provided written informed consent before participating.

## Supplementary Information

Below is the link to the electronic supplementary material.

Supplementary file1 (DOCX 18 KB)

## Data Availability

Upon request, and subject to review, Pfizer will provide the data that support the findings of this study. Subject to certain criteria, conditions, and exceptions, Pfizer may also provide access to the related individual deidentified participant data. See https://www.pfizer.com/science/clinical-trials/trial-data-and-results for more information.
