# Supplementary material for: Pan-Asian subgroup analysis of EV-302/KEYNOTE-A39: a phase 3 study to evaluate enfortumab vedotin and pembrolizumab in patients with untreated advanced urothelial carcinoma
Source: Int J Clin Oncol. 2026 Jan 21;31(3):436–46. doi: 10.1007/s10147-025-02950-8 (PMC12932347; doi:10.1007/s10147-025-02950-8)
Supplement: Supplementary file 1 — Supplementary file1 (DOCX 18 KB) [file 10147_2025_2950_MOESM1_ESM.docx]

# Supplementary Material

## **Online Resource 1** Summary of subsequent systemic therapies: pan-Asian subgroup

| **Subsequent therapy, n (%)** | **Enfortumab vedotin–pembrolizumab (*n* = 94)** | **Chemotherapy (*n* = 82)** |
| --- | --- | --- |
| Patients who received subsequent systemic therapies^a^ | 21 (22.3) | 54 (65.9) |
| First subsequent systemic therapy |  |  |
| Platinum-based therapy^b^ | 18 (19.1) | 8 (9.8) |
| PD-1/PD-L1 inhibitors | 1 (1.1) | 42 (51.2) |
| Maintenance therapy^c^  Avelumab  Pembrolizumab | 0  0  0 | 18 (22.0)  17 (20.7)  1 (1.2) |
| Second-line therapy  Atezolizumab  Nivolumab  Pembrolizumab | 0  0  0  1 (1.1) | 24 (29.3)  9 (11.0)  1 (1.2)  14 (17.1) |
| Other | 2 (2.1) | 4 (4.9) |

^a^Twenty-one of 94 patients (22.3%) in the enfortumab vedotin–pembrolizumab group were still receiving the treatment as of data cutoff date of August 8, 2024.

^b^When platinum-based therapy and a PD-1/PD-L1 inhibitor were given in the same line of therapy, the therapy was categorized under platinum-based therapy.

^c^Patients could have received more than one PD1/PD-L1 inhibitor in the same line of therapy.

PD-1, programmed cell death protein 1; PD-L1, programmed death ligand 1.

## **Online Resource 2** Enfortumab vedotin treatment-related adverse events of special interest^a^

| **Treatment-related adverse events, *n* (%)** | **Enfortumab vedotin–pembrolizumab** **(*n* = 94)** | | **Chemotherapy (*n* = 76)** | |
| --- | --- | --- | --- | --- |
|  | **Any grade** | **Grade ≥3** | **Any grade** | **Grade ≥3** |
| Skin reactions^b^ | 77 (81.9) | 27 (28.7) | 13 (17.1) | 0 |
| Peripheral neuropathy  Sensory events  Motor events | 62 (66.0)  62 (66.0)  3 (3.2) | 8 (8.5)  8 (8.5)  0 | 12 (15.8)  12 (15.8)  0 | 0  0  0 |
| Hyperglycemia | 20 (21.3) | 10 (10.6) | 0 | 0 |
| Ocular disorders  Dry eye | 8 (8.5)  6 (6.4) | 0  0 | 0  0 | 0  0 |
| Infusion-related reactions | 0 | 0 | 0 | 0 |

^a^Patients were included from the safety analysis set which consisted of all patients randomly assigned who received at least 1 dose of investigational product (or any component of combination therapy).

^b^There were differences in the rates of skin reactions reported for enfortumab vedotin treatment-related AEs of special interest and pembrolizumab treatment-emergent AEs of special interest, because these AEs were reported via different methodologies developed for enfortumab vedotin and pembrolizumab monotherapies, respectively.

AE, adverse events.

## **Online Resource 3** Pembrolizumab treatment-emergent adverse events of special interest^a^

| **Treatment-emergent adverse events, *n* (%)** | **Enfortumab vedotin–pembrolizumab** **(*n* = 94)** | | **Chemotherapy (*n* = 76)** | |
| --- | --- | --- | --- | --- |
|  | **Any grade** | **Grade ≥3** | **Any grade** | **Grade ≥3** |
| Severe skin reactions^b^ | 21 (22.3) | 17 (18.1) | 0 | 0 |
| Pneumonitis | 16 (17.0) | 7 (7.4) | 0 | 0 |
| Hypothyroidism | 16 (17.0) | 2 (2.1) | 1 (1.3) | 0 |
| Hyperthyroidism | 4 (4.3) | 0 | 0 | 0 |
| Gastritis | 2 (2.1) | 0 | 0 | 0 |
| Adrenal insufficiency | 3 (3.2) | 0 | 0 | 0 |
| Myositis | 2 (2.1) | 0 | 0 | 0 |
| Thyroiditis | 2 (2.1) | 0 | 0 | 0 |
| Hepatitis | 1 (1.1) | 0 | 1 (1.3) | 0 |
| Cholangitis sclerosing | 1 (1.1) | 1 (1.1) | 0 | 0 |
| Pancreatitis | 1 (1.1) | 1 (1.1) | 1 (1.3) | 1 (1.3) |
| Myocarditis | 1 (1.1) | 0 | 0 | 0 |
| Optic neuritis | 1 (1.1) | 0 | 0 | 0 |

^a^Patients were included from the safety analysis set which consisted of all patients randomly assigned who received at least 1 dose of investigational product (or any component of combination therapy).

^b^There were differences in the rates of skin reactions reported for enfortumab vedotin treatment-related AEs of special interest and pembrolizumab treatment-emergent AEs of special interest, because these AEs were reported via different methodologies developed for enfortumab vedotin and pembrolizumab monotherapies, respectively.

AE, adverse event.
